# Association of octacosanol supplementation with redox status in patients on chronic statin therapy

**DOI:** 10.5937/jomb0-38224

**Published:** 2023-01-20

**Authors:** Milica Zrnić-Ćirić, Jelena Kotur-Stevuljević, Ivan Stanković, Brižita Đordjević, Ivana Baralić, Miodrag Ostojić

**Affiliations:** 1 University of Belgrade, Faculty of Pharmacy, Department of Bromatology, Belgrade; 2 University of Belgrade, Faculty of Pharmacy, Department of Medical Biochemistry, Belgrade; 3 Zvezdara University Medical Center, Belgrade; 4 University of Belgrade, Faculty of Medicine, Belgrade; 5 Institute for Cardiovascular Diseases "Dedinje", Belgrade + University Clinical Centre of the Republic of Srpska, Banja Luka, Bosnia and Herzegovina + University of Banja Luka, Medical Faculty, Banja Luka, Bosnia and Herzegovina

**Keywords:** dyslipidaemia, atorvastatin, octacosanol, redox status, dislipidemije, atorvastatin, oktakozanol, redoks status

## Abstract

**Background:**

The uneven lipid-lowering statin effects and statin intolerance raise interest regarding the involvement of coadministration of statins and dietary supplements. This study aimed to evaluate the effects of octacosanol supplementation on markers of redox status in cardiovascular patients on chronic atorvastatin therapy.

**Methods:**

A double-blind, randomized, placebo-controlled, single-centre study was conducted. Redox status homeostasis parameters [i.e., advanced oxidation protein products (AOPP), pro-oxidant-antioxidant balance (PAB), total oxidant status (TOS), total antioxidant status (TAS), superoxide dismutase activity (SOD), total protein sulfhydryl (SHgroups), and paraoxonase 1 (PO N 1) activity] were assessed in 81 patients. According to favorable changes in lipid profile, patients were classified into two groups: responders (n = 35) and non-responders (n = 46), and followed for 13 weeks. A principal component analysis (PCA) was applied to explore the effect of octacosanol supplementation and the relationship between investigated parameters as predictors of responders' and non-responders' status.

**Results:**

Significant decrease in Oxy-score value was found at the endpoint compared to baseline in responders' group (21.0 (13.4-25.5) versus 15.1 (12.4-18.0); *P* < 0.01). PCA analysis extracted 4 significant factors in the both groups, whereas extracted factors containing "octacosanol status" variable explained 14.7% and 11.5% of the variance in responders' and non-responders' subgroups, respectively.

**Conclusions:**

Octacosanol supplementation leads to an improvement of lipid profile and markers of redox status in responders' group. New studies are needed to validate our results in order to find the best approach for personalized supplementation as a useful adjunct to standard statin therapy.

## Abstract (sr)

**Uvod:**

Neujednačeni hipolipemijski efekti primene statina kao i intolerancija na statine dovode do porasta inte resovanja u vezi sa zajedničkom primenom statina i dijetetskih suplemenata. Ova studija je imala za cilj da proceni efekte suplementacije oktakozanolom na markere redoks statusa kod kardiovaskularnih pacijenata na hroničnoj terapiji atorvastatinom.

**Metode:**

Sprovedena je dvostruko slepa, randomizovana, placebo kontrolisana studija. U serumu 81 pacijenta određivani su parametri redoks statusa [produkti uznapredovale oksidacije proteina (AOPP), prooksidativno-antioksidativni balans (PAB), totalni oksidativni status (TOS), totalni antioksidativni status (TAS), aktivnost superoksid dismutaze (SOD), sulfihidrilne grupe (SH-grupe) i aktivnost paraoksonaze 1 (PON1)]. U odnosu na povoljne promene lipidnog profila, pacijenti su klasifikovani u dve grupe: responderi (n = 35) i neresponderi (n = 46), i praćeni su 13 nedelja.

**Rezultati:**

Značajno smanjenje vrednosti oksi-skora primećeno je kod respondera na kraju studije u poređenju sa vrednostima na početku studije (21,0 (1 3,4 -25,5) vs. 15,1 (12,4 -18,0); P < 0,01). Principal component analiza (PCA) je primenjena da bi se procenio efekat suplementacije oktakozanolom kao i odnos između ispitivanih parametara kao prediktora odgovara u obe grupe. PCA analiza je izdvojila po 4 značajna faktora u obe grupe, pri čemu faktori koji sadrže varijablu "status oktakozanola" objašnjavaju 14,7% i 11,5% ukupne varijacije kod respondera i nerespondera, redom.

**Zaključak:**

Suplementacija oktakozanolom dovodi do poboljšanja lipidnog profila i markera redoks statusa u grupi respondera. Buduće studije su potrebne za potvrdu dobijenih rezultata a u cilju pronalaženja optimalnog pristupa za personalizovanu suplementaciju uz standardnu terapiju statinima.

## Introduction

Cardiovascular disease (CVD) continues to be the leading cause of morbidity and mortality worldwide [1]. Although there are many different causes of CVD, the association between CVD and oxidative stress (OS) is well documented [2]. OS is defined as an imbalance between the production of oxidative compounds, including reactive oxygen species (ROS), and endogenous antioxidant defense systems, in favour of the former [3]. ROS are normally produced during the metabolism through the mitochondrial electron transfer chain or by redox reactions and are necessary components for cellular homeostasis. The revised definition of OS not only comprises the abovementioned imbalance but also the shift in redox signaling that may have both positive and harmful effects [4]. The interaction of ROS with numerous biomolecules leads to the formation of a variety of products which are often referred as biomarkers of OS [5]. Excessive levels of ROS promote direct and irreversible oxidative damage to macromolecules, as well as disruption of redox-dependent cell signaling mechanisms [2].

Statins are among the most commonly prescribed medicines worldwide for the prevention of CVD mainly due to lowering the concentration of low-density lipoprotein cholesterol (LDL-C), but the effectiveness of statins therapy is also related to their pleiotropic activities [6]. The ability to reduce OS by modulating redox systems is an important mechanism by which statins exert beneficial effects on the cardiovascular system [7] [8]. The clinical significance of the pleiotropic effects of statins, independent of the lipidlowering actions, remains controversial [9], referring to more clinical trials which would establish the relevance of these effects.

Studies conducted both in vitro and *in vivo* support the antioxidant role of statins in the development of atherosclerosis [10] and CVD [11], but data on statins' antioxidative effects from human intervention studies are ambiguous [12] [13] [14] [15]. However, many studies indicate that statin-induced OS is a plausible mechanism for adverse effects associated with their use such as various diabetic complications [16], myopathy [17] and the development of fatty liver [18]. Statins may exert protective effects against OS in atherosclerotic tissues, but they also may cause hepatotoxicity, nephrotoxicity and muscle toxicity by OS [19]. Different pleiotropic effects of statins have gained increasing attention, as well as antioxidants used as antagonists in the toxicities of statins. Coadministration of statins with antioxidants from natural plants may obtain a putative synergistic benefit and reduce the adverse effects related to statins.

Octacosanol is the main component of policosanol, a mixture of long-chain primary fatty alcohols separated and purified from sugar cane, rice bran, wheat and beeswax. A meta-analysis reported that policosanol could be used to decrease LDL-C [20]. Policosanol decreases 3-hydroxyl-3-methylglutaryl coenzyme A (HMG-CoA) reductase activity and inhibits the synthesis of cholesterol in cultured cells [21] [22] and animals [23] which may be a potential mechanism for lowering cholesterol levels. The inhibition is related to the activation of adenosine monophosphate protein (AMP)-kinase concomitant with phosphorylation of HMG-CoA reductase [24]. It is very important to point out that the activation of AMP-kinase is sustained by oxidation of very long chain fatty alcohols, that makes up policosanol, to fatty acids. Moreover, in intervention studies, policosanol supplementation resulted in blood pressure decreasing by increased high-density lipoproteincholesterol (HDL-C) [25] [26]. Interestingly, it has been suggested that policosanol may prevent the rise of proprotein convertase subtilizing/kexin type-9 (PCSK9) levels in patients who start statin therapy, as well as mildly reduce PCSK9 in healthy volunteers [27]. PCSK-9 facilitate the LDL-receptor degradation and compromised LDL-C clearance in the circulation [28]. In addition to its lipid-lowering effect, policosanol showed *in vitro* antioxidant, antiglycation and antiapoptotic effects which all could enhance HDL functionality [29]. However, there are limited data on the effects of octacosanol on redox status *in vivo*. Recent results indicated that octacosanol may reduce OS in athletes during strength training [30]. Taken together, octacosanol may reduce lipid peroxidation and improve the antioxidant capacity of mitochondria and prevent myocardial damage [31].

In our previously published study [32], we used the strategy of sub-dividing the participants into groups according to changes in lipid parameters that have already made the distinction of the participants into responders and non-responders. Thus, in this randomized double-blind, placebo-controlled study, we aimed to investigate the potential of standard biochemical analysis and parameters of redox status and antioxidant defense to predict the responsiveness to supplement usage of octacosanol in patients on chronic statin therapy.

## Materials and methods

### Study design and patients

A randomized, double-blind, placebo-controlled parallel-groups study was conducted following the guidelines laid down in the Declaration of Helsinki. The trial procedure was approved by the ethics committee of University Clinical Centre Zvezdara, Belgrade, Serbia. Written consent from all participants was obtained before initiating the study. The trial was registered at Australian New Zealand Clinical Trials Registry (ACTRN12619000102178; http://www.anzctr.org.au).

One-hundred and seventy-seven outpatients from the Department of Cardiology, University Clinical Centre Zvezdara, Belgrade, Serbia were initially screened for eligibility. Finally, eighty-seven men and women, aged 40–80 years, were recruited for this 13-week study. All patients had a diagnosis of hypercholesterolemia or mixed dyslipidaemia with a minimum four-month using atorvastatin (20 mg/day) prior to study. The power calculation has been done before the study and was described in detail in our previous report [32]. Patients with acute coronary syndrome(within the previous month), serious heart failure, renal dysfunction, liver disorders, cerebral vascular disease or mental illness were excluded from the study. None of the participants used substances or practiced habits known to act pro-oxidative or affect anti-oxidative defense system. During 13 weeks of intervention period, participants did not change their individual dietary habits and preferences. Participants were randomly assigned to receive either atorvastatin 20 mg/d + placebo (*n* = 45) or atorvastatin 20 mg/d + dietary supplement (DS) which contains Octa cosanol (20 mg) and Vitamin K2 (40 μg) (*n* = 42). All capsules (DS and placebo) were supplied by Abela Pharm d.o.o., Belgrade, Serbia. Placebo capsules were identical to DS capsules in terms of their size, color, shape, and smell. The placebo contained all the ingredients of DS capsules except for the bioactive ingredients. Participants were scheduled for follow-up visits when any unusual adverse effects were reported by using appropriate record forms. To monitor compliance, patients were asked to return any unused capsules.

### Baseline and clinical data collection

Information about health conditions on studied participants was obtained from their medical records and in cooperation with their physicians. Prior application of complete clinical history, the following data were collected at baseline: demographics (age, gender, height, and weight) and whether they smoked or not as well as measurement of subjects’ blood pressure. Body mass index (BMI) for each individual was calculated using body weight (kg) divided by the square of the height (m).

### Sample collection and biochemical analyses

Twelve-hour fasting blood samples were collected between 9 and 10 a.m. at baseline and at the end of 13-week supplementation period. Venous blood was collected into one serum vacutainer and one EDTA vacutainer tube (Vacutainer; Becton, Dickinson and Company, NYSE, USA), separated by centrifugation (1500 x g, 15 min) and multiple aliquots of each sample were stored at -80°C until analysis of antioxidant/pro-oxidant status markers and PCSK9 levels.

Biochemical parameters were determined on the same day using clinical chemistry analyser(Hitachi 7150, Tokyo, Japan) with commercial kits (Boehringer, Mannheim, Germany). The following parameters were determined: total cholesterol (TC), HDL-cholesterol (HDL-C), LDL-cholesterol (LDL-C), triglycerides (TG), apolipoprotein A1 (ApoA1), and apolipoprotein B100 (ApoB100), as well as aspartate aminotransferase (AST), alanine aminotransferase (ALT), creatine phosphokinase (CK), C-reactive protein (CRP), and glucose.

Total serum PCSK9 concentration was measured in duplicate using a commercial, high-sensitivity, quantitative sandwich enzyme immunosorbent assay (Human PCSK9 Quantikine ELISA, R&D Systems Inc., Minneapolis, USA).

We assessed several anti-oxidative and pro-oxidative parameters, as specific markers of free radicaldamaging activity. All these spectrophotometric methods were implemented at ILAB 300 Plus analyser (Instrumentation Laboratory, Milan, Italy). As markers of anti-oxidative status, total antioxidative status (TAS), sulphydryl (SH) groups, serum paraoxonase (PON1) and superoxide dismutase activity (SOD) were estimated. TAS was measured by a colorimetric test using 2,2-azino-bis(3-etilbenzotiazolin)-6-sulfonic acid (ABTS) as a chromogen [33]. The colour intensity proportionally decreases to the total concentration of all antioxidants in the sample. The concentration of SH groups was determined by the method of Ellman using 2,2’-dinitro-5,5’-dithio-benzoic acid in an alkaline medium [34]. Serum PON1 activity was measured kineticallyusing paraoxon as substrate, whereby it convert to *p-*nitrophenol, by method of Richter and Furlong [35]. Serum SOD activity was determined according to modified method of Misra and Fridovich [36], based on the ability of the enzyme to inhibit spontaneous autooxidation of epinephrine. SOD activity was calculated as a percent of inhibition of epinephrine autooxidation.As markers of redox status, we measured total oxidative status (TOS), prooxidative-antioxidative balance (PAB) and advanced oxidation protein products (AOPP). TOS was assessed by a spectrophotometric method optimized by Erel [37], with ferrous ion o-dianisidine as a chromogen. Total oxidants in the sample oxidize this chromogen to a ferric ion which then forms a coloured product in a reaction with xylenol-orange. The results were expressed as H_2_O_2_ equivalents which was used as a standard. PAB was determined by a modified assay [38] using 3, 3’, 5, 5’-tetramethylbenzidine and its cation as a redox indicator. PAB was expressed in arbitrary HK units (HKU), which correspond to the percentage of hydrogen peroxide in the standard solution. AOPP were measured by spectrophotometry after reaction with acetic acid and potassium-iodide in phosphate buffer pH 7.4 [39].

In addition, the Oxy-score was calculated by subtracting the antioxidative score (an average of standardized antioxidant variables TAS and SHG) from the prooxidative score (an average of standardized markers of redox status variables) [40].

### Statistical analyses

The Shapiro-Wilk’s test was used to determine the type of data distribution. Data are shown as mean otherwise, as geometric mean (standard deviation) for normally distributed variables, and 95^th^ confidence intervals for log-transformed values, or median and interquartile range for variables not normally distributed. Categorical variables were presented as absolute and relative frequencies. Subjects’ baseline characteristics were tested with independent-sample t test for normally distributed parameters and Mann–Whitney U-test for parameters that were with non-normal distribution. Paired Student’s t-test or nonparametric Wilcoxon’s two related samples test was used for evaluation of parameters in the same group. Chi-squaredtest was used to analyse the differences in categorical data. Correlation between tested parameters was investigated by Spearman correlation analysis. Principal component analysis-PCA (varimax-normalized rotation) was implemented to reduce the number of examined variables to a smaller number of factors according to its level of variability. Data included in this analysis was either normally distributed variables or variables with non-normal distribution, after log transformation. Extracted factor was selected if its eigenvalues >1. Variables with factor loadings >0.5 were used for the analysis. Analyses were performed using the SPSS software (ver. 20.0; Chicago, IL) and P values < 0.05 were deemed to indicate statistical significance.

## Results

We selected favourable changes in lipid profile, defined as absolute changes in LDL-C and and HDL-C levels (decrease and increase, respectively) after 13 weeks of antihyperlipidemic therapy with atorvastatin alone or with atorvastatin/octacosanol combination, as the criteria for classifying patients as responders and non-responders. Accordingly, patients were classified into two groups: (1) responders (n = 35) (participants changes in lipid profile defined as decrease in LDL-C and increase in HDL-C) and (2) non-responders (n = 46) (participants who did not reach expected therapy goals). Six participants who dropped out before the end of the study were not included in the final data analysis.

The anthropometric and clinical characteristics of responders and non-responders are listed in Table 1. Gender distribution, body mass index and blood pressure data were similar in both groups. Responders had a much higher prevalence of smoking (34.3%) than non-responders (15.2%). There were no significant differences in clinical characteristics between the two groups, except the use of diuretics.

**Table 1:** BMI, Body mass index; ACE, Angiotensin-converting enzyme<br>Continuous variables are presented as mean (standard deviation), unless otherwise specified (^‡^ median (interquartile range)); categorical variables are presented as absolute and relative frequencies. Continuous variables were compared by Student t test or by Mann-Whitney exact test, and categorical variables by Chi-square test.

Parameter	Non-responders (n = 46)	Responders (n = 35)	*p*
Age, years	61.9 (8.4)	63.1 (7.1)	0.511
Gender, female/male	17/29	13/22	0.986
BMI, kg/m^2^	27.2 (4.0)	28.2 (3.3)	0.262
Prevalence of smoker, n (%)	7(15.2)	12 (34.3)	0.045
Systolic blood pressure, mm Hg^‡^	130 (115–140)	130 (120–140)	0.276
Diastolic blood pressure, mm Hg^‡^	80 (80–80)	80 (80–80)	0.142
Diabetes mellitus, n (%)	9 (19.6)	11 (31.4)	0.220
Family history of coronary heart disease, n (%)	21 (45.7)	24 (68.6)	0.200
Previous myocardial infarction, n (%)	26 (56.5)	21 (60.0)	0.269
Atorvastatin, n (%)	22 (47.8)	22 (62.9)	0.179
Atorvastatin + octacosanol, n (%)	24 (52.2)	13 (37.2)	
Cardioprotective medications			
Beta-blocker, n (%)	38 (82.6)	29 (82.9)	0.977
Calcium channel blocker, n (%)	14 (30.4)	8 (22.9)	0.448
Diuretics, n (%)	28 (60.9)	11 (31.4)	0.009
Antianginal drugs, n (%)	21 (45.7)	17 (48.6)	0.794
Aspirin/clopidogrel, n (%)	40 (87)	32 (91.4)	0.526
ACE inhibitors, n (%)	39 (84.8)	31 (88.6)	0.622

Results of standard biochemical analyses including lipid profile of the participants are displayed in Table 2. At baseline, average HDL-C levels were significantly higher in non-responders (*p *< 0.05), while LDL-C was increased (*p* < 0.05) in responders. Obtained changes in HDL-C and LDL-C levels are attributable to the criteria for subdividing participants. Other biochemical parameters were similar in both investigated groups. In addition, CRP levels were higher in responders compared with non-responders (*p* < 0.05). Results of parameters indicating redox status and antioxidant defense status for all responders and non-responders are also presented in Table 2. Regarding the parameters for redox status at baseline, the responders’ group had a higher value of prooxidant–antioxidant balance than in non-responders (*p* < 0.05). Also, TAS levels were lower in responders compared to the non-responders (p < 0.05) of TOS and SHG were noticed in responders’ group (p=0.057 and p=0.070, respectively), while AOPP levels were higher compared to non-responders’ group (p=0.066), although with marginal statistical significance.

**Table 2:** Standard biochemical analysis and parameters of redox status and antioxidant defense in responders and non-responders at baseline and after 13th week. TC, total cholesterol; HDL-C, high-density lipoprotein cholesterol; LDL-C, low-density lipoprotein cholesterol; TG, triglycerides; ApoA1: Apolipoprotein A1; ApoB100: Apolipoprotein B100; PCSK9: Proprotein convertase subtilisin/kexin type 9; CRP: C-reactive protein; ALT, alanine aminotransferase; AST, aspartate aminotransferase; CK, creatine phosphokinase; AOPP, advanced oxidation protein products; PAB, prooxidant antioxidant balance; SHG, sulphydryl groups; SOD, superoxide dismutase; TAS, total antioxidant status; TOS, total oxidant status; PON1, paraoxonase1. Data are presented as mean (standard deviation), unless otherwise specified (†geometric mean values (95th CI), ^‡^ median (interquartilerange)). a Paired Student’s t-test or nonparametric Wilcoxon two related samples test were used for evaluation of parameters in the same group(responders and non-responders, respectively, 13th week vs. baseline comparison). b Variables among different groups (responders vs. non-responders) at two time points, respectively, were compared using Student’s t test (parametric data) or Mann–Whitney U test (nonparametric data). ^a, b^P < 0.05; aa, ^bb^P < 0.01;^ aaa^P < 0.001.

Parameter	Non-responders (n = 46)		Responders (n = 35)	
	Baseline	13th week	Baseline	13th week
Glucose	5.44 (5.00–6.05)	5.74 (5.25–6.79)	5.97 (5.16–6.53)	6.01 (5.42–7.13)
TC, mmol/L	4.49 (1.09)	4.87 (1.16)^aa^	4.75 (1.02)	4.61 (0.96)
HDL-C, mmol/L	1.44 (0.41)	1.40 (0.38)	1.28 (0.28)^b^	1.59 (0.35)^aaa^
LDL-C, mmol/L	2.45 (0.98)	2.91 (1.09)^aa^	2.94 (0.94)^b^	2.23 (0.90)^aaa^
TG, mmol/L^†^	1.28 (1.14–1.44)	1.27 (1.11–1.47)	1.40 (1.17–1.67)	1.53 (1.30–1.82)
ApoA1, g/L	1.45 (0.25)	1.46 (0.27)	1.44 (0.25)	1.40 (0.23)
ApoB100, g/L	1.80 (0.50)	1.98 (0.57)a	1.93 (0.48)	1.97 (0.49)
PCSK9, ng/mL^†^	212 (181–248)	225 (192–263)	215 (182–253)	203 (158–261)
CRP, mg/L^†^	1.28 (1.04–1.58)	1.55 (1.25–1.91)	1.90 (1.39–2.59)^b^	2.12 (1.67–2.70)
ALT, U/L^†^	21 (20–22)	19 (17–21)	21 (19–24)	21 (19–23)
AST, U/L^†^	23 (22–25)23.47	20 (19–22)^aa^	24 (22–26)23.82	21 (20–23)^aa^
CK, U/L^†^	110 (97–124)	109 (91–130)	95 (73–123)	94 (78–114)
TAS, mmol/L^‡^	1078 (513–1464)	1652 (1122–1785)^aaa^	532 (465–931)^b^	1108 (910–1732)^aaa^
SOD, U/L	109 (27)	103 (18)	110 (31)	107 (24)
SHG, mmol/L^‡^	0.300 (0.200–0.500)	0.500 (0.400–0.500)^aaa^	0.200 (0.100–0.400)	0.400 (0.300–0.500)^aaa^
PON1, U/L^†^	286 (222–369)	329 (113–526)^a^	249 (190–326)	220 (154–314)
TOS, mmol/L^‡^	7.5 (6.0–12.0)	17.2 (16.1–18.3)^aaa^	6.0 (5.0–9.0)	16.0 (11.0–19.0)^aaa^
AOPP, μmol/L	24.8 (7.3)	25.2 (7.2)	28.6 (10.8)	24.5 (6.3)^a^
PAB, HKU	113 (42)	131 (46)^a^	132 (35)^b^	123 (41)

After 13 weeks of intervention period, as shown in Table 2, significantly increased concentrations ofTAS and SHG (*p* < 0.001) were observed in both groups. In addition, it was noted that PON1 activity was significantly increased only in non-responders’ group (*p* < 0.05). With regard to markers of redox status, AOPP was significantly decreased (*p* < 0.05) in responders’ group at the end of the study compared to the baseline levels, while PAB value was significantly elevated (*p* < 0.05) in non-responders. TOS levels were increased towards the end of the study in both groups (*p* < 0.001).

**Figure 1:**
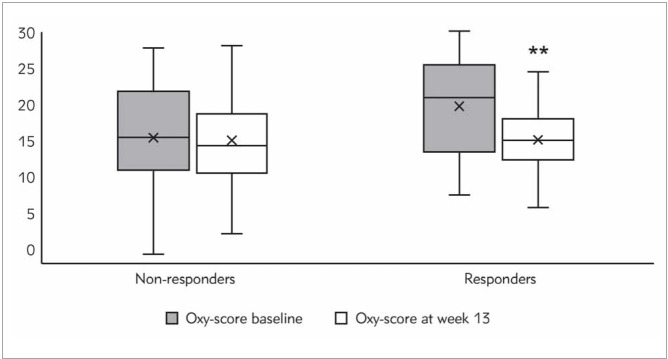
Oxy-score in non-responders and responders at the baseline and after 13^th^ week of intervention period.

Calculated values of Oxy-score in both groups are presented in Figure 1. The initial values of Oxy-score were significantly higher in responders’ group compared to non-responders’ group (20.97 (13.44–25.47) vs. 15.46 (10.95–21.82); *P* < 0.05). The analysis revealed a significant Oxy-score decrease at the endpoint compared to baseline in responders’ group (20.97 (13.44–25.47) versus 15.07 (12.36–18.04); *P* < 0.01).

PCA analysis was implemented on all investigated parameters. Regarding the non-responders’ group of parameters Kaiser-Meyer-Olkin measure of sample adequacy parameter (0.562) and Bartlett’s sphericity tests (p<0.001) confirmed the selected model adequacy. The whole model showed 55.1% of total variability and the analysis extracted 4 factors which we have entitled: Dyslipidemia-redox balance factor (TOS, TAS, PON1, PAB and PCSK9), Lipid risk-inflammation protein related factor (TG, CRP, SHG and ApoB100), Metabolic-related lipid protective factor (ALT, AST and ApoA1) and Intervention-related metabolic antioxidant factor (SOD, glucose and octacosanol supplement status – yes/no). For the responders’ group of parameters PCA analysis showed adequacy measures as follows: Kaiser-Meyer-Olkin adequacy parameter (0.573) and Bartlett`s sphericity tests (p<0.01), which confirmed model adequacy. Factorial analysis extracted 4 significant factors (Dyslipidemia-redox balance inflammation related factor (TOS, TAS, PAB, PCSK9 and CRP), Coronary risk intervention-related factor (Total CK activity, ALT, AST and octacosanol supplement status – yes/no), Lipid risk-protein related redox factor (AOPP, SHG and ApoB100) and Metabolic-related antioxidant factor (ApoA1, Glucose and PON1)) which explained 55.0% of the total variance. Detailed data from PCA analysis was showed at the Table 3.

**Table 3:** Factor loadings for examined parameters that loaded highly (| > 0.5|) in varimax rotated principal components for non-responders and responders^a^. TG, triglycerides; ApoA1: Apolipoprotein A1; ApoB100: Apolipoprotein B100; PCSK9: Proprotein convertase subtilisin/kexin type 9; CRP: C-reactive protein; ALT, alanine aminotransferase; AST, aspartate aminotransferase; CK, creatine phosphokinase; AOPP, advanced oxidation protein products; PAB, prooxidant antioxidant balance; SHG, sulphydryl groups; SOD, superoxide dismutase; TAS, total antioxidant status; TOS, total oxidant status; PON1, paraoxonase1. ^a^Only variables with factor loadings | ≥ 0.5| are displayed and listed in order for simplicity and easy of interpretation. ^b^Non-responders’ factors; Dyslipidemia-redox balance factor: TOS, TAS, PON1, PAB and PCSK9; Lipid risk-inflammation protein related factor: TG, CRP, SHG and ApoB100; Metabolic-related lipid protective factor: ALT, AST and ApoA1; Intervention-related metabolic antioxidant factor: SOD, glucose and octacosanol supplement. ^c^Responders’ factors; Dyslipidemia-redox balance inflammation related factor: TOS, TAS, PAB, PCSK9 and CRP; Coronary risk intervention-related factor: Total CK activity, ALT, AST and octacosanol supplement; Lipid risk-protein related redox factor: AOPP, SHG and ApoB100; Metabolic-related antioxidant factor: ApoA1, Glucose and PON1

	Non-responders^b^				Responders^c^			
	Factor 1	Factor 2	Factor 3	Factor 4	Factor 1	Factor 2	Factor 3	Factor 4
Factor variability (%)	16.6	14.7	12.2	11.5	15.5	14.7	12.2	12.1
PON1	-0.631	-	-	-		-	-	0.668
PCSK9	0.509	-	-	-	0.565	-	-	-
TAS	0.766	-	-	-	0.789	-	-	-
TOS	0.829	-	-	-	0.681	-	-	-
PAB	0.608	-	-	-	0.537	-	-	-
CRP	-	0.576	-	-	-0.526	-	-	-
TG	-	0.792	-	-	-	-	-	-
ApoB100	-	0.678	-	-	-	-	0.629	-
SHG	-	0.589	-	-	-	-	0.661	-
AOPP	-	-	-	-	-	-	0.829	-
ApoA1	-	-	0.560	-	-	-	-	0.727
AST	-	-	-0.632	-	-	0.650	-	-
ALT	-	-	-0.645	-	-	0.785	-	-
Total CK activity	-	-	-	-	-	0.800	-	-
Octacosanol supplement –<br>yes/no	-	-	-	-0.728	-	-0.619	-	-
SOD	-	-	-	0.607	-	-	-	-
Glucose	-	-	-	0.608	-	-	-	0.675

## Discussion

A wealth of data demonstrates a causal link between serum LDL-C levels and CVD. The currently available HMG-CoA reductase inhibitors (statins) and PCSK-9 inhibitors have shown great promise in achieving LDL-C therapeutic target. Since their lifelong prescriptions prompt various side effects, nutraceuticals with dual HMG-R and PCSK-9 inhibitory activities have been extensively studied these days [41] [42]. In addition to HMG-CoA inhibitory activity, it has been also shown that policosanol may reduce circulating levels of PCSK9 in the animal model [43] as well as in healthy participants [27].

In the present study, we have evaluated the response of several standard lipid status biomarkers, PCSK9, markers of redox status and antioxidant defense on the standard antihyperlipidemic therapy (atorvastatin) and combined therapy (atorvastatin + octacosanol). All participants were subdivided into responders’ or non-responders’ groups according to absolute changes in lipid parameters (LDL-C decrease and HDL-C increase). Results regarding the beneficial effects of a 13-week octacosanol supplementation on PCSK9 levels in patients on chronic statin therapy have been published in our previous report [32] and may be referred to for more detailed information not related to this article.

We found that LDL-C levels of responders were significantly lower (p<0.001) after 13 weeks of the intervention period. Moreover, it was observed that average LDL-C was significantly lower in non-responders compared to responders at the beginning of the study. Interestingly, these results indicate that beneficial effects are most prominent in individuals with higher baseline levels of LDL-C. Concomitant significant decrease of AST activity in both groups of patients speaks in favour of statin pleiotropic effects.

OS is an unifying hallmark of all CVD risk factors as well as a factor that is elevated in response to neurohormonal activation in heart failure [44]. In this study, levels of pro-oxidants (TOS) significantly increased in both examined groups as well as levels of antioxidants (TAS and SHG) compared to baseline values, while PON1 increased only in non-responders’ group. An increased antioxidative capacity in all participants after the therapy period might be partly explained as a consequence of pleiotropic statin and octacosanol effects or as a compensatory mechanism linked to elevated mitochondrial ROS production and apoptosis. Although the complex mechanism of statin-induced mitochondrial dysfunction is not fully understood, it is proposed that atorvastatin may increase mitochondrial ROS formation and induce apoptosis [45]. AOPP and TOS were independently correlated with HDL-C in non-responders’ and responders’ groups, respectively, whereas no independent correlation between other markers of redox homeostasis and lipid parameters were found.

Individual redox-related biomarkers in human CVDs only partially describe the redox status and are associated with a large intra- and inter-subject variability [40]. Since complex results were obtained regarding redox status parameters, we calculate Oxy-score, a recently developed indicator of OS that combines individual biomarkers of oxidative damage and antioxidant capacity, to assess the overall oxidative balance in CVDs [46] [47]. Regarding Oxy-score values, a statistically significant decrease was observed in responders’ group, while there was no change in the non-respondersgroup. It is important to note that responders had a higher Oxy-score value at baseline compared to nonresponders. Given these results at the end of the study, responders not only have improved lipid profile but also have decreased the Oxy-score value. Recently, Quintana-Villamandos et al. [48] have demonstrated that Oxy-score could help in the diagnosis of clinical cardiovascular manifestations and also could be used to monitor treatment response.

To further explore the effect of octacosanol supplementation and the interrelationship between different parameters of lipid profile, OS and other risk factors as predictors of responders’ and non-responders’ status, PCA was applied. This analysis identified four different factors in examined groups explaining very similar total percent of the explainable variation, 55.1% and 55% in non-responders’ and responders’ groups, respectively. The PCA-derived factor 1 explained 16.6% and 15.5% of the total variance in non-responders and responders, respectively, and interestingly it was associated with positive loadings of the same four variables: PCSK9, TAS, TOS and PAB. PON1 activity and CRP additionally characterised Factor 1 in non-responders’ and responders’ groups,respectively. Although Factor 1 demonstrated consistencies in both groups, some differences were also acknowledged in the other identified factors. PCA demonstrates different values of factor variability between PCA extracted factors containing »octacosanol status« variable in investigated groups. The obtained results support the notion that octacosanol supplementation could contribute to overall effects in responders’ group. Factor 2 explained 14.7% of the variance and consisted of Coronary risk interventionrelated factor (total CK activity, ALT, AST and octacosanol supplement) in responders’ group. On the contrary, in non-responders’ group PCA-derived factor 4 explained 11.5% of the variance and consisted of Intervention-related metabolic antioxidant factor (SOD, glucose and octacosanol supplement). Interestingly, in responders’ group, PCA analysis combined »octacosanol status« and the markers of statins’ side effects. Liver damage, muscle pain, and increased risk of type 2 diabetes mellitus are some of the side effects of statins. Octacosanol when used with background long-term atorvastatin therapy would mitigate some of these side effects [31] [49].

Clarity regarding whether policosanol (octacosanol) supplementation may be useful for lipid dysmetabolism is still lacking. Early studies of policosanol supplementation performed by Cuban researchers showed reductions in both TC and LDL-C levels [49] [50], while these results could not be confirmed by other studies [51]. A recent meta-analysis comprising 22 studies reported that policosanol could be used to lower lipid content and to elevate HDL-C levels [20]. Nevertheless, policosanols are components of dietary supplements recommended for the prevention as well as cotreatment of lipid dysmetabolism and related CVD.

As far as authors are aware, data on effects of octacosanol on redox marker in patients treated with statins are unknown. Most studies have explored the effects of policosanol (octacosanol) in subjects under physiological conditions when their natural antioxidant defences are not compromised or challenged. Arteche-Hidalgo et al. [52] have investigated the effects of policosanol when the participants are exposed to increase OS due to pathological conditions it found that 6 months treatment with policosanol in patients with metabolic syndrome reduce the redox index. In addition, Lee et al. [30] found that octacosanol may be a beneficial supplement to improve antioxidant enzyme levels in athletes after high-intensity training.

New approaches in designing intervention studies, including sub-dividing participants into responders and non-responders, as well as analysing the results, suggest the identification of specific markers able to predict the individual response to dietary supplements [53]. It is tempting to promote the use of dietary supplements specifically to potential responders and avoid it in potential non-responders. In this sense, based on the inter-individual differences that may occur in response to examined treatment, obtained results have distinguished the subjects that can benefit from octacosanol supplementation from those that cannot have any advantages from added supplement to chronic atorvastatin therapy.

Our study had certain limitations. Since the sample size of this study is considered a relatively small cohort in terms of intervention studies, future investigation consisting of a larger cohort, could validate these results. The absence of statin naïve participants limited the potential for detailed statistical analyses, but our results provide a picture of a real-life scenario of added supplement to long-term atorvastatin use. We used this particular supplement because it was available as an over-the-counter product. All the available data indicates that low-dose vitamin K2 (45 μg) does not have any lipid regulating effects. A 3-month of supplementation is the most commonly used period. Future studies may also consider observing the effects of octacosanol supplementation at different time points along longer supplementation periods and/or at various dosing regimen. Octacosanol lipophilicity inhibits solubility in water, resulting in poor absorption and low bioavailability, thus delaying its health-promoting effects.

The novelties of the study should also be emphasized. To the best of our knowledge, there areno studies that performed PCA to evaluate the joint effect of various cardiometabolic parameters and redox-related biomarkers on patients’ response to a combination using atorvastatin and octacosanol. Moreover, no previous studies examined Oxy-score values in patients with chronic atorvastatin use, and these results may contribute to better understand differences between antihyperlipidemic therapy responders and non-responders.

## Conclusion

The study results indicate that octacosanol supplementation may promote favourable cardioprotective changes, when added to atorvastatin therapy, by improvements of lipid profile and markers of redox status in the responders’ group. This may be particularly helpful in patients who cannot tolerate high-dose statin therapy. However, it would not be advisable to omit statin therapy without considering dose lowering and the inclusion of supplements. Future studies may help identify patient profiles, linked to genetic variants of PCSK9 polymorphisms, who are likely to respond to personalized supplementation as a useful adjunct to standard pharmacotherapy.

## Dodatak

### Acknowledgements

This work was supported by the Ministry of Education, Science and Technological Development of Serbia on the basis of contract No.451-03-68/2022-14/200161.

### Conflict of interest statement

All the authors declare that they have no conflict of interest in this work.
